# Predicting priority management areas for land use/cover change in the transboundary Okavango basin based on machine learning

**DOI:** 10.1016/j.heliyon.2023.e22762

**Published:** 2023-11-23

**Authors:** Blessing Kavhu, Zama Eric Mashimbye, Linda Luvuno

**Affiliations:** a Department of Geography and Environmental Studies, Stellenbosch University, Private Bag X1, Matieland, 7602, South Africa; b Centre for Sustainability Transitions, Stellenbosch University, Stellenbosch, 7600, South Africa; c Scientific Services Unit, Zimbabwe Parks and Wildlife Management Authority, Headquarters, P. O. Box 140, Causeway, Harare, Zimbabwe

**Keywords:** Landcover trajectories, Water resources, Transboundary basin, Post-Angolan war, Machine learning, Ensemble modelling

## Abstract

Remote sensing and modelling of land use/land cover (LULC) change is useful to reveal the extent and spatial patterns of landscape changes at various environments and scales. Predicting susceptibility to LULC change is crucial for policy formulation and land management. However, the use of machine learning (ML) for modelling LULC change is limited. This study modelled LULC change susceptibility in the Okavango basin using ML techniques. Areas with high LULC change susceptibility are termed priority management areas (PMAs) in this study. Trajectories of LULC change between 1996 and 2020 are derived from existing LULC change maps of the Okavango basin. Overlay analysis is then used to detect patches of LULC change transitions. Three LULC transitional categories are adopted for modelling PMAs, namely 1) from natural to anthropogenic classes (Category A); 2) from anthropogenic to natural classes (Category B); and 3) from natural to another natural class (Category C). An ensemble of ML algorithms is calibrated with categories of LULC change and social-ecological drivers of change to produce maps showing the susceptibility of LULC change in the basin. Thereafter, thresholding is done on probability maps of susceptibility to LULC change based on the maximum sum of sensitivity and specificity (max SSS) to delineate PMAs. Results for trajectories of LULC change indicate that anthropogenic activities (croplands, built-up areas, and barelands) generally expanded, displacing natural areas (wetlands, woodlands, water, and shrubland) from 1996 to 2020. Regarding PMAs, anthropogenic-related PMAs (Category A ∼34 560 km^2^) covered a larger area compared to the natural ones (Categories B∼33 407 km^2^) and (Categories C∼15 040 km^2^). The findings of this study emphasize the value of ensemble ML modelling in identifying PMAs and guiding transboundary land use planning. Overall, this study highlights the role of anthropogenic activities in driving land use changes in Transboundary Drainage Basins (TDBs) and suggests a need to promote sustainable practices in predicted PMAs through comprehensive planning to ensure water availability in the Okavango basin.

## 1 Introduction

Remote sensing and modelling of land use/cover change (LULC) is crucial for policy formulation, natural resources, and land management. Globally, LULC change is largely driven by anthropogenic activities, climate change, and natural disasters [1,2]. Change in LULC can have significant impacts on social-ecological systems (SES) and livelihood support systems, as it threatens ecosystem services provision. As human populations expand and urbanize, agricultural practices intensify, and infrastructure development accelerates, LULC transformations become inevitable [[3], [4], [5]]. Such alterations can disrupt ecosystem services, alter hydrological cycles, fragment habitats, and compromise biodiversity, consequently impacting the livelihoods and well-being of communities that depend on these systems [6,7]. For instance, according to INPE [8], the Amazon rainforest reported an increase in deforestation rates by 140 % between 2020 and 2021, contributing to a net loss of 13 200 km^2^ of forest cover. This shift in land use not only resulted in the release of approximately 2.6 gigatons of carbon dioxide into the atmosphere but also disrupted the traditional livelihoods of indigenous communities and threatened biodiversity. Similarly, according to the United Nations [9], urbanization has led to significant land transformation, with estimates showing that by 2050, the urban population is expected to double, causing challenges related to water availability, air quality, and social inequalities. These reported trends underscore the urgency of understanding and managing LULC change to maintain the delicate balance between ecological sustainability and human well-being. The use of remote sensing and modelling for LULC change monitoring would be crucial.

Remote sensing has emerged as a powerful tool for unveiling the dynamics and consequences of LULC changes across diverse landscapes. For instance, in a study by Aksoy et al. [10], remote sensing data in the form of an urban atlas and CORINE data were employed to assess LULC alterations in Eskişehir, revealing a notable surge in artificial surfaces compared to natural ones. Similarly, Cetin et al. [11] utilized remote sensing, specifically NDVI differencing, to gauge the impact of newly established universities on urban expansion in Turkey, detecting a significant 4.49 % growth in certain university areas. In another study by Cetin [4], remote sensing was employed using a LULC change-based approach to delineate research priority areas and socio-economic drivers of development along the coastal region of Cide in Turkey. Moving to the Southern Hemisphere, Münch et al. [12] employed remote sensing, focusing on LULC changes and surface albedo analysis, to assess catchment water and carbon fluxes in South Africa's Eastern Cape Province. While it is evident that remote sensing can provide relevant information to track previous LULC change (trajectories) and its impacts, effective monitoring requires future projections based on modelling. Thus, integrating remote sensing and models could aid in developing plans for the sustainable management of resources.

Modelling LULC change is useful to reveal the extent and spatial patterns of anticipated changes at various scales. For purposes of policy formulation and resource management, the results of LULC modelling can expose areas that require prioritization. According to Verburg et al. [13], LULC change modelling can range from local to global scales. The drivers of LULC change will vary, depending on the scale. So will the policies applicable at various scales and the decision that can be made based on the LULC modelling results.

Transboundary environments are unique and delicate settings in which the LULC changes are influenced by a complex mix of different policies governing land management, different cultures involved, and contestation that may exist in certain environments [14,15]. Modelling LULC change at a transboundary scale is a unique analysis that can reveal crucial information to inform policies for sustainable management and use of shared resources [16,17]. Given the complexity of transboundary environments, effective modelling is essential to guide monitoring efforts with robust and easily interpretable results. However, existing techniques often struggle to strike a balance between these two aspects. Wang et al. [18] categorized modelling techniques into cellular automata (CA) and machine learning (ML) methods. CA, a traditional approach, evolves based on predefined rules and includes models like SLEUTH and Conversion of Land Use and its Effects (CLUE) [19,20]. ML techniques, on the other hand, learn from data and make informed decisions, and include algorithms such as support vector machine, random forest (RF), and k-nearest neighbor [21].

Recent studies have favoured ML techniques due to their reported robustness [22,23]. However, ML's probability map outputs often confuse land managers, especially when faced with defining the threshold for distinguishing highly susceptible from less susceptible areas. This presents an opportunity to propose the use of thresholding techniques, like the maximum sum of sensitivity and specificity (max SSS), available in ML algorithms [24]. These techniques can generate binary output maps from probability maps, highlighting areas that are highly susceptible to LULC change (hereafter referred to as priority management areas (PMAs)) that enable ease of monitoring and planning for expansive regions such as TDBs. Wang et al. [18] extensively reviewed studies that used ML models to predict LULC changes. They established that ML and DL are limited in modelling LULC change because of: 1) lack of spatio-temporal transition mechanisms to describe occurrence, transition, and spatial patterns of changes; 2) lack of training data of all the drivers of change and; 3) unavailability of inclusion of local ecological, hydrological, and socio-economic drivers when assessing the spectral feature changes. Apart from that, we did not find any study that targeted distinctly delineating high and less susceptible areas of LULC for purposes of prioritization and planning. Also, previous studies often relied on single modelling approaches, which could be limiting in complex environments such as TDBs. These are critical gaps that need to be filled to improve the presentation of model outputs to land managers and aid decision-making in complex environments such as TDBs.

The Okavango basin is a prime example of a key SES that has been threatened by LULC change in the past decade, attributed to various factors including increased anthropogenic activities [25]. After the end of the war in Angola in 2002, there has been increased pressure on resources in some parts of the basin [26]. This calls for urgent solutions to combat potential ecological catastrophe and ensure the sustainable management of the basin's resources. One of the possible solutions lies in the development of strategic management practices that are guided by maps of PMAs for the basin. Delineation of PMAs offers the ability to monitor, analyze, and predict LULC changes. However, the development of effective PMAs requires an analysis based on unparalleled precision and efficiency such as those offered by remote sensing and spatial modelling [27,28].

Previous research successfully established baseline data on LULC change using remote sensing in the Okavango basin. For instance, VanderPost [29], made an initial attempt to classify LULC classes in the basin, but their study had a significant limitation in that it only covered the 10 km buffer zone along major rivers and tributaries. This certainly excluded crucial information from other areas of the basin. Another study by Andersson [30], evaluated the LULC of the entire basin, but it focused solely on the period during the Angolan Civil War (1971–2001). Given the post-war political changes and developments that could impact the SES in the basin, it has been necessary to assess LULC changes during the post-war period. Although Kavhu et al. [31], tested the most accurate technique for mapping LULC for the entire basin for the period during the Angolan War and post-war period, what is missing in literature is information on the trajectories of LULC change during the post-war period and the identification of PMAs in the Okavango basin. These are important information gaps that need to be closed to develop some strategic land use plans for the Okavango basin. Evaluating PMAs for LULC change in the resource-constrained Okavango basin would enable maximum management impact using minimal effort [32].

To address the earlier mentioned gaps, this study aims to assess the trajectories of LULC change and model PMAs at a transboundary scale using ML approaches. The analysis was conducted for the period between 1996 and 2020 for the Okavango basin. LULC transitions are categorized into three categories, namely anthropogenic to natural, natural to anthropogenic, and natural to natural classes. The susceptibility to LULC change is modelled first and areas that are highly susceptible to change are considered to be prioritized by land managers and for policy formulation. These areas are referred to as PMAs in this study. This study aims to guide land management in identifying areas that could require prioritization to inform policy. Also, we think that the investigation could affirm the detection and modelling of LULC change in complex settings such as transboundary environments using ML.

## 2 Materials and methods

### 2.1 Description of the study site

**Fig. 1:**
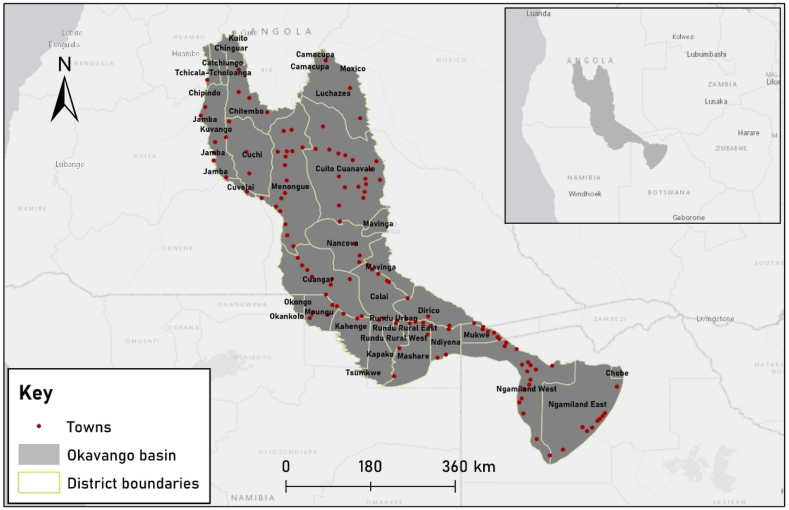
Study area map showing locations of towns and districts of the Okavango basin.

The study was conducted in the Okavango basin, spanning a latitude range between 12°S and 21°S and a longitude range between 16°E and 24°E, as depicted in Fig. 1. This transboundary region extends across three nations, namely Angola, Namibia, and Botswana. The Okavango basin experiences a semi-arid climate characterized by seasonal rainfall and predominantly hot, dry conditions. Summers frequently bring about droughts, which in turn create challenges related to high temperatures and access to surface water across much of the basin.

While the average annual temperature ranges around an annual average of 20 °C, it undergoes significant fluctuations, leading to pronounced evapotranspiration levels that affect surface water systems [33]. One of the most remarkable features of the Okavango basin is its possession of the largest inland delta in the world, known as the Okavango Delta, a UNESCO World Heritage site [34]. Additionally, the Okavango River ranks as the fourth longest river system in Southern Africa and exhibits a distinctive annual flooding pattern [35]. These flooding patterns hold paramount importance for both the livelihoods of local communities within the basin and the region's diverse wildlife species. Communities residing in the Okavango basin have adapted their agricultural practices to synchronize with these annual flooding cycles, a method known as Molapo crop farming [36].

### 2.2 Methods

**Fig. 2:**
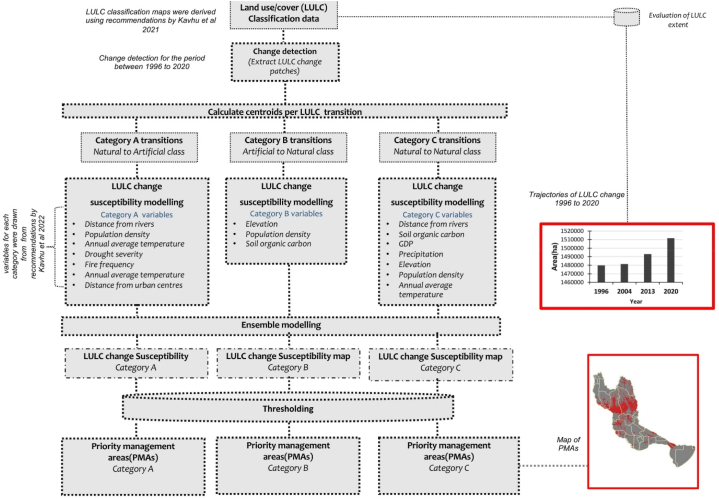
Study workflow.

This study incorporates the results of two investigations we conducted in the Okavango basin, namely Kavhu et al. [31] and Kavhu et al. [37]. An improved multitemporal LULC classification of the Okavango basin generated using a deep neural network (DNN) that incorporated spectral bands, vegetation indices, and regionalization based on the Köppen-Geiger's climate zones laid the foundation for this investigation [31]. Social-ecological drivers of LULC change were then characterized with the improved LULC dataset using ML, of which LULC change transitions were categorized [37]. Thereafter, centroids of LULC change transitions and social-ecological covariates were used to model LULC susceptibility using machine learning (ML). The study workflow in Fig. 2 summarizes the procedure that was followed in this investigation. Brief descriptions of these methods are provided in the succeeding subsections.

#### 2.2.1 Multitemporal LULC classification

Multitemporal LULC maps were generated using analysis-ready Landsat 5 and Landsat 8 OLI images acquired in June for the years 1996, 2002, 2013, and 2020. A combined integration of climate-based regionalization using Köppen-Geiger climate zones, spectral bands, and spectral indices to enhance the accuracy of multitemporal land use/cover classification using deep learning and ML approaches was investigated based on two experiments. While the first experiment entailed the integration of spectral bands with spectral indices, the second involved the combined integration of spectral indices and climate-based regionalization using analysis-ready Landsat 5 TM and Landsat 8 OLI images. ML (RF and extreme gradient boosting) and deep learning (neural network and DNN) classifiers using supervised classification with a total of 5140 samples were used to classify multitemporal LULC for the years 1996, 2004, 2013, and 2020. The samples were split into training and validation. The study established that a combination of spectral indices, climate-based regionalization, and recursive feature selection significantly enhanced the accuracy of multitemporal LULC classification for all classifiers. However, the classification-based DNN yielded the most accurate multitemporal LULC classification. Hence, the LULC classification for this analysis was based on a DNN.

#### 2.2.2 LULC change mapping

To identify LULC change, a post-classification change analysis was conducted by overlaying LULC maps for two time steps: 2004–2013 and 2013–2020. A change matrix was compiled by examining the intersection of different pairs of LULC maps. Patches and centroids of LULC transitions were generated using ArcMap 10.8. To classify LULC change transitions in a manageable way for land managers and transboundary commissions, the categorization scheme introduced by Kavhu et al. [37] was employed. This scheme includes the following transitions: natural-artificial classes (Category A), artificial-natural classes (Category B), and natural-natural classes (Category C). By adopting this categorization scheme, the computational complexity of analyzing individual transitions is reduced. Table 1 provides a comprehensive list of the categories and their corresponding LULC change transitions.

**Table 1:** Categories of LULC transitions as adopted from Kavhu et al. [37].

Category	List of transitions
**Category A**	water – cultivated woodland – built-up woodland – cultivated grassland – built-up grassland – cultivated shrubland – cultivated wetland – cultivated
**Category B**	cultivated – built-up cultivated – woodland cultivated – grassland cultivated – shrubland
**Category C**	water – woodland water – grassland woodland – grassland woodland – shrubland grassland – water grassland – shrubland grassland – wetland shrubland – woodland shrubland – grassland shrubland – wetland wetland – woodland wetland – grassland wetland – shrubland

#### 2.2.3 Modelling LULC change PMAs

An ensemble of ML algorithms – incorporating presence data (centroids of LULC change patches) and a set of social-ecological covariates to assess the susceptibility of LULC change – was employed.

The selection of LULC change transitions for modelling susceptibility to LULC change focused on the post-war period from 2004 to 2013. This approach aimed to capture changes influenced by the current social-ecological conditions in the basin and maintain consistency with the other investigations we conducted in the basin. Ten social-ecological variables obtained from diverse remote sensing data sources were used. The variables included measures such as distance from rivers, population density, fire frequency, annual average temperature, drought severity, distance from urban centers, elevation, soil organic carbon, gross domestic product, and precipitation. Further details on the acquisition, pre-processing, and modelling procedures for the data can be found in Kavhu et al. [37].

The ensemble of ML algorithms comprised RF, gradient boost models, maximum entropy (MaxEnt), classification tree analysis, and artificial neural network. The combined dataset (presence and absence) was split, with 80 % used for model training and 20 % for model testing per run. The performance of the models was evaluated using the true skill statistic (TSS) and the receiver operating curve (ROC). A ROC or TSS value below 0.5 indicates a poor model, while values within the range of 0.5–0.8 represent a good model. ROC values exceeding 0.8 indicate excellent model performance [38].

The final outputs from the modelling process were binary maps, indicating areas highly susceptible to LULC change and those deemed non-susceptible. A threshold of ROC ≥0.7 was utilized to create a binary map based on max SSS as recommended by Liu et al. [24]. Although continuous maps could have depicted the potential susceptibility of LULC change, the binary map was employed to delineate areas with high susceptibility to LULC change. Areas with high susceptibility to LULC change in this investigation were designated as areas that require prioritization by land managers, so-called PMAs. These were then masked using a boundary layer for districts to identify administrative areas that fall under PMAs.

## 3 Results and discussion

### 3.1 Trajectories of LULC classes

**Fig. 3:**
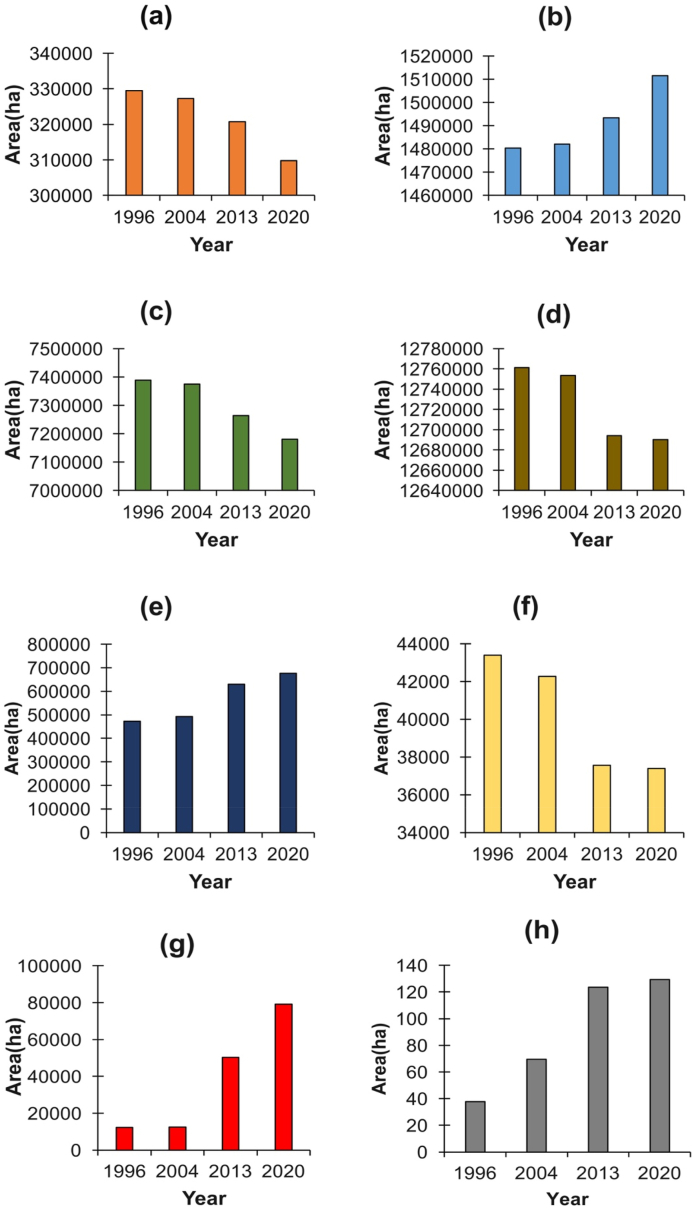
Trends in the extent of LULC classes during the years 1996, 2004, 2013 and 2020 for the following classes: (a)wetland, (b)grassland, (c)shrubland, (d)woodland, (e)cropland, (f)water, (g)built-up, (h)bareland

**Fig. 4:**
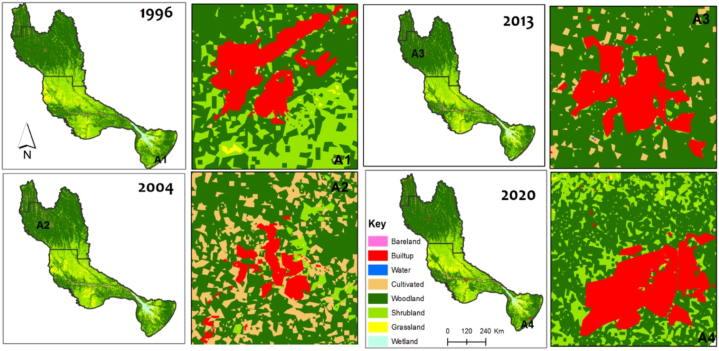
Changes in the extent and distribution of LULC classes during the years 1996, 2004, 2013 and 2020. Subfigures A1,A2,A3,A4, shows changed LULC classification results at different times and the respective satellite view based on high-resolution imagery as adopted from [31].

Fig. 3, Fig. 4 show the LULC dynamics within the Okavango basin over 24 years, spanning from 1996 to 2020. This trend reveals the significant transformation in the basin's natural landscape. Notably, there is a pronounced decline in the extent of natural cover, including crucial natural classes such as wetlands (from 330 000 ha to 310 000 ha), shrublands (from 7 390 876 ha to 7 119 889 ha), woodlands (from 12 760 000 ha to 12 690 000 ha), water bodies (from 43 000 ha to 37 000 ha), and (Fig. 3 a, c, d & f). This decline is most likely linked to the effect of various anthropogenic activities. Specifically, there is a significant increase in the extent of croplands (from 500 000 ha to 700 000 ha) and built-up areas (from 10 000 ha to 80 000 ha) within the basin (Fig. 3e and g). This indicates that anthropogenic activities have played a pivotal role in the evolution of LULC in the Okavango basin. These results are in line with observations by McCarthy and Ellery [39] and Mendelsohn and El Obeid [33] who recorded that the cessation of the Angolan War appears to have triggered a post-war population rebound. This demographic resurgence likely exerted pressure on the region's resources, leading to heightened demands for settlement areas, agricultural land, grazing grounds for livestock, and timber for fuel and construction purposes. As a direct consequence, we observed the expansion of anthropogenic LULC classes such as built-up areas, croplands, and barelands. This expansion is accompanied by the depletion of shrublands and woodlands (Fig. 3c and d). The increase in grasslands over the period is surprising (Fig. 3b). Generally, grasslands have been recorded to be threatened by settlement expansion and the effects of climate change [40]. This study's observation that grasslands are increasing is likely a consequence of misclassification. Fallow lands have probably been misclassified as grasslands, leading to the observation that it is increasing.

The implications of these shifts in LULC are far-reaching. As the extent of woodlands, shrublands, wetlands, and water bodies dwindle, the ecological balance of the basin is disrupted. This not only affects the region's biodiversity but also has profound implications for its hydrology, as water resources are increasingly stressed. Furthermore, it is essential to acknowledge the role of climate-related threats in this complex interplay. The Okavango basin has experienced a series of environmental challenges, including droughts, floods, and fires, as reported by Neuenschwander [41] and Byakatonda et al. [42] These extreme events further exacerbate the vulnerability of the natural landscape, compounding the pressure induced by anthropogenic activities. The findings of this study suggest the adoption of a multidimensional approach to address the observed LULC dynamics in the Okavango basin. This will involve policy decisions, land management strategies, and conservation efforts that consider the complex interplay of factors contributing to these changes. For instance, solutions should not merely focus on trying to mitigate the impacts of anthropogenic activities but also on building resilience in the face of climate-related threats. This could go a long way in safeguarding the ecological integrity and surface water availability in the Okavango basin.

Findings of this study on the expansion of anthropogenic classes at the expense of natural classes in the Okavango basin are not new. Previous studies have observed an increase in cropland extent and a decrease in water extent in the same landscape during the period before and during the Angolan War [29,30]. However, unlike previous studies, this study focused on the post-Angolan War period. The strength of this study lies in not just identifying LULC trends but in constructing comprehensive trajectories of LULC changes during a critical time frame. The LULC maps of the post-Angolan War period offer a nuanced understanding of the current relationship between anthropogenic activities and natural resources in the basin. This exposes the complex impacts of increased anthropogenic developments on the region's ecology. Besides, this will also catalyse more robust discussions and actions towards sustainable management of water resources in this TDB.

### 3.2 LULC change susceptibility modelling

**Fig. 5:**
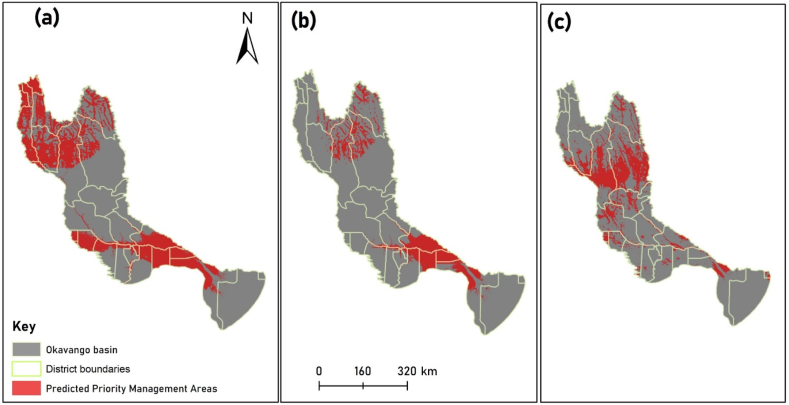
The spatial extent of predicted PMAs for LULC change within and across administrative districts in the Okavango basin; (a) natural – anthropogenic transition, (b) anthropogenic – natural transition (c) natural – natural transition.

**Fig. 6:**
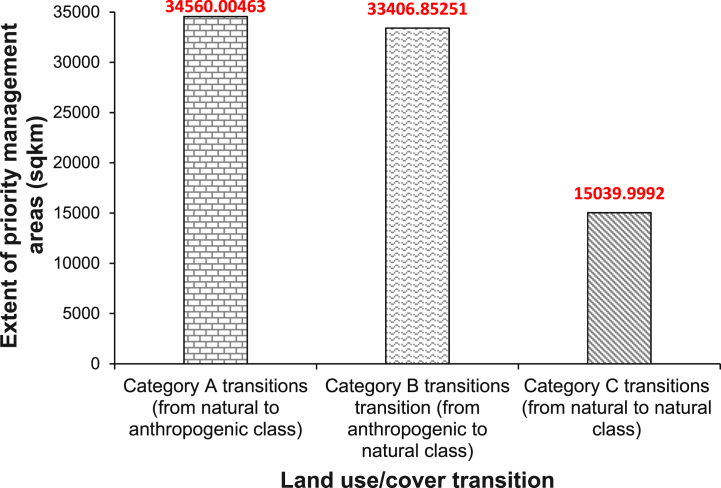
The extent of PMAs of LULC change for different categories of change transitions.

Reliable results for high susceptibility of LULC based on ML were obtained in this study. Our ensemble model successfully predicted areas that are highly susceptible to LULC change (PMAs) for Category A (ROC = 98.3, TSS = 92.4), Category B (ROC = 97.6, TSS = 90.1), and Category C (ROC = 96.2, TSS = 91.3). The predicted extent of PMAs for LULC change within and across administrative districts in the Okavango basin varies with the category of transition and country. Fig. 5, Fig. 6 show the extent of PMAs for LULC change in the basin for the three categories.

The predicted high susceptibility to LULC change (termed PMAs in this study) for Category A transitions (Fig. 5a) spanning three countries within the Okavango basin, encompassing a total of 31 districts (Angola – 19, Botswana – 2, Namibia – 10), is a significant outcome of this study (please refer to Appendix 1 for a comprehensive list of districts). These PMAs are distributed across various geographical regions, including the central, southern, and northwestern parts, with a particular emphasis on areas like Menongue in Angola, Rundu in Angola, and Ngamiland West in Botswana. These findings hold implications for land managers and transboundary commissions who are tasked with managing and regulating anthropogenic activities in the Okavango basin. It underscores the urgent need to prioritize resources and conservation efforts in these identified areas. Notably, this study corroborates the previous work of Andersson [30], who identified similar areas at risk from anthropogenic activities, specifically Rundu, Menongue, and Cuito Cuanavale. However, the current study extends beyond previous studies by identifying 29 additional districts that were not previously reported as PMAs. The strength of this study lies in its comprehensive and up-to-date approach, as it is based on results of the complete extent of the Okavango basin, utilizing recent satellite imagery spanning from 2004 to 2020. This is in contrast to Andersson [30], who focused solely on the northern part of the basin, relying on older imagery from 1973 to 2001. The advantage of employing complete and up-to-date distribution maps becomes evident in this study, as it was able to reveal the presence of PMAs in districts previously unexplored by earlier research. This underscores the evolving nature of environmental dynamics in the Okavango basin and the necessity for adaptable management strategies. As such, findings of this study not only affirm the importance of existing conservation efforts but also emphasize the need for a dynamic and responsive approach to safeguarding the ecological integrity of this vital region.

The predicted PMAs for Category B transitions (Fig. 5b) show a diverse spatial distribution, spanning 24 districts within the basin, with notable concentrations in the northeastern and southern regions. Particularly dominant are the extensive areas in Menongue and Cuito Cuanavale in Angola, as well as Ngamiland West in Botswana (for a comprehensive list of districts, please refer to Appendix 2). These regions have experienced significant disruptions primarily due to anthropogenic activities, especially agriculture. Given this insight, it becomes apparent that these areas may demand minimal investment when initiating agro-successional restoration projects, aligning with the findings. For example, Török et al. [43] emphasized the importance of a cost-effective approach to restoration, specifically through the promotion of spontaneous succession by minimizing further human interventions. Vieira et al. [44] recommended that agro-successional restoration models should be prioritized, offsetting management costs, while providing food security for small landholders and involving small landholders in the restoration process. It is therefore crucial to adopt a more nuanced perspective when practicing restoration projects in these areas.

Regarding Category C transitions (Fig. 5c), the projected PMAs are concentrated in northeastern districts, encompassing Cuito Cuanavale and Luchazes in Angola (please refer to Appendix 3 for district details). This spatial distribution appears to be influenced by lingering landmine issues in Angola, which have historically hindered development and anthropogenic activities in this particular region. Vanderpost et al. [29], attributed underdevelopment in Northern Angola to the presence of landmines which presents difficulties for human settlement. The limited anthropogenic influence in such areas may facilitate natural processes, driving LULC transitions within Category C. The findings of this investigation guide transboundary commissions and local authorities in potential prioritization of these regions when crafting intervention strategies aimed at mitigating the effects of natural processes such as droughts, floods, invasions, and fires. However, it should be noted that the absence of anthropogenic activities does not necessarily equate to resilience. These areas may still be vulnerable to external factors and require careful planning to ensure sustainable management. Furthermore, an interesting observation emerged when considering Categories, A and B transitions, where PMAs overlap in certain districts within the northern and southern sectors of the basin, such as Menongue in Angola and Ngamiland West in Botswana, respectively (Fig. 5 a & b). This coexistence offers a chance for integrated monitoring and management approaches that could result in economical solutions.

Fig. 6 shows that the spatial extent of Category A (∼34 560 km^2^) transitions in the PMAs surpasses that of Categories B (∼33 407 km^2^) and C (∼15 040 km^2^). This observation not only underscores the distinct influence of anthropogenic factors on LULC changes but also prompts careful consideration of the implications. The findings of this study are consistent with studies elsewhere by Gupta and Sharma [45], Thonfeld et al. [46] and Tian et al. [47]. These studies consistently highlighted the role of anthropogenic activities in driving LULC changes. Notably, the rapid expansion of anthropogenic developments differs from natural processes, such as ecological succession. Anthropogenic activities can spread faster, leading to rapid changes in large land tracts [[48], [49], [50]]. This distinction highlights the urgency and scale of the challenges posed by anthropogenic activities in the landscape. Furthermore, the adaptive capacity of human populations is a critical factor to consider. As reported by Burke et al. [51], humans have an exceptional ability to adapt to changing environments, creating new opportunities for their diverse practices. This adaptability raises important questions about the sustainability and long-term consequences of LULC changes in the Okavango basin.

This study presents novel insights into the existing body of knowledge. While previous research has acknowledged the impact of anthropogenic activities in the Okavango basin, the extent and distribution of areas that should be prioritized for managing these impacts remained largely unknown. Thus, this study is among the first to generate spatially explicit data on areas of high LULC change susceptibility (∼PMAs) for monitoring and management in the Okavango basin. The implications of this study's findings are multifaceted. It offers a crucial foundation for planning and policy formulation; opportunities to identify PMAs for the strategic allocation of resources and interventions to manage the expansion of anthropogenic activities effectively; and also serves as a valuable resource for ensuring the equitable distribution of water resources in the Okavango basin, aligning with broader goals of sustainable water management and environmental conservation.

## 4 Conclusions

This study investigated an ML-based method for modelling PMAs of LULC change in a complex transboundary environment. LULC change transitions were categorized into three categories, namely natural to artificial, artificial to natural, and natural to natural. The susceptibility of LULC change was then modelled using an ensemble of ML approaches based on LULC change transitions and socio-ecological variables.

The study revealed that the extent of anthropogenic classes such as croplands, built-up areas, and barelands increased over 20 years, while natural classes like wetlands, woodlands, water, and shrubland decreased. Although grasslands were also observed as having increased in extent, we think that this is unlikely. This finding is likely due to misclassification. The PMAs were successfully modelled using ML algorithms and subsequent thresholding based on max SSS. We found that transitions from anthropogenic to natural classes were larger than those of anthropogenic to natural, and natural to natural. This highlights the dominance of anthropogenic activities in driving LULC change in the basin areas that could require attention, identified using districts in the Okavango basin.

Overall, the study concludes that ML techniques can be used to delineate PMAs in TDBs and identified PMAs for the Okavango basin, offering promising prospects for restoration and intervention. Furthermore, a more critical perspective acknowledges the potential consequences of anthropogenic activities, the complexities of regions affected by landmines, and the need for holistic, integrated approaches in areas of coexistence. Sustainable land management in these different areas will necessitate a delicate balance between restoration efforts and respecting the inherent resilience of natural processes. Apart from generating empirical evidence of human-driven LULC changes, this study calls for a more critical examination of the societal and ecological consequences of the observed changes. The complex interplay between rapid anthropogenic developments, human adaptability, and environmental sustainability requires careful consideration as we chart the future course for the Okavango basin. Furthermore, the study emphasizes the need for a dynamic and responsive approach to safeguarding the ecological integrity of this transboundary basin.

## Data availability statement

The training data utilized in this study can be made available upon request from the authors, provided that consent is granted by certain data providers. The authors are currently not authorized to publicly share some of the training data from OKACOM and NGOWP unless permission is obtained.

## Funding statement

The authors thank the USAID Resilient Waters Project for supporting this research under prime contract number 72067418C00007.

## CRediT authorship contribution statement

**Blessing Kavhu:** Writing – review & editing, Writing – original draft, Visualization, Validation, Methodology, Investigation, Formal analysis, Data curation, Conceptualization. **Zama Eric Mashimbye:** Writing – review & editing, Writing – original draft, Validation, Supervision, Methodology, Conceptualization. **Linda Luvuno:** Writing – review & editing, Writing – original draft, Validation, Supervision, Resources, Methodology, Conceptualization.

## Declaration of competing interest

The authors declare that they have no known competing financial interests or personal relationships that could have appeared to influence the work reported in this paper.

## Figures and Tables

**Appendix 1:** List of districts that contain PMAs for Category A transitions

Angola	Botswana	Namibia
Camacupa	Ngamiland East	Kahenge
Chinguar	Ngamiland West	Kapako
Chitembo		Mashare
Kuito		Mpungu
Calai		Mukwe
Cuangar		Ndiyona
Cuchi		Rundu Rural East
Cuito Cuanavale		Rundu Rural West
Dirico		Okongo
Mavinga		Okankolo
Menongue		
Cuvelai		
Chipindo		
Jamba		
Kuvango		
Catchiungo		
Tchicala-Tcholoanga		
Luchazes		
Moxico		
**19**	**2**	**10**

**Appendix 2:** List of districts that contain PMAs for Category B transitions

Angola	Botswana	Namibia
Camacupa	Ngamiland East	Kahenge
Chinguar	Ngamiland West	Kapako
Chitembo		Mashare
Calai		Mpungu
Cuangar		Mukwe
Cuchi		Ndiyona
Cuito Cuanavale		Rundu Rural East
Dirico		Rundu Rural West
Mavinga		
Menongue		
Nancova		
Catchiungo		
Luchazes		
Moxico		
**14**	**2**	**8**

**Appendix 3:** List of districts that contain PMAs for Category B transitions

Angola	Botswana	Namibia
Camacupa	Ngamiland East	Kahenge
Chitembo	Ngamiland West	Kapako
Calai		Mashare
Cuangar		Mpungu
Cuchi		Mukwe
Cuito Cuanavale		Ndiyona
Dirico		Rundu Rural East
Mavinga		Rundu Rural West
Menongue		Okongo
Nancova		Okankolo
Cuvelai		Grootfontein
Jamba		Tsumkwe
Kuvango		
Luchazes		
Moxico		
Chobe		
**16**	**2**	**12**
